# Biotechnology Potential of Marine Fungi Degrading Plant and Algae Polymeric Substrates

**DOI:** 10.3389/fmicb.2018.01527

**Published:** 2018-07-10

**Authors:** Larissa Balabanova, Lubov Slepchenko, Oksana Son, Liudmila Tekutyeva

**Affiliations:** ^1^G.B. Elyakov Pacific Institute of Bioorganic Chemistry, Far Eastern Branch, Russian Academy of Sciences, Vladivostok, Russia; ^2^Innovative Technology Center, Far Eastern Federal University, Vladivostok, Russia

**Keywords:** filamentous fungi, marine-derived fungi, glycoside hydrolases, algae polysaccharides, plant polysaccharide-degrading enzymes, lignocellulolytic enzymes

## Abstract

Filamentous fungi possess the metabolic capacity to degrade environment organic matter, much of which is the plant and algae material enriched with the cell wall carbohydrates and polyphenol complexes that frequently can be assimilated by only marine fungi. As the most renewable energy feedstock on the Earth, the plant or algae polymeric substrates induce an expression of microbial extracellular enzymes that catalyze their cleaving up to the component sugars. However, the question of what the marine fungi contributes to the plant and algae material biotransformation processes has yet to be highlighted sufficiently. In this review, we summarized the potential of marine fungi alternatively to terrestrial fungi to produce the biotechnologically valuable extracellular enzymes in response to the plant and macroalgae polymeric substrates as sources of carbon for their bioconversion used for industries and bioremediation.

## Introduction

Marine fungi are widely distributed microorganisms in the ocean, particularly associated with sediment, seawater, marine habitants, submerged plants, and algae. Currently, culture-based analyses and genomic sequencing have identified 1112 marine fungal species in 472 genera. The *Halosphaeriaceae* is the largest family of marine fungi, while the genera *Aspergillus*, *Penicillium*, and the yeast genus *Candida* are most widespread (Le Calvez et al., 2009; Jones et al., 2015; Kumar et al., 2015; Richards et al., 2015). However, the sequence similarity-based approach continues to reveal the fungal taxonomic classification that should adequately reflect their ecology and chemical potential (Reich and Labes, 2017). The fungal life cycle and mediating interactions between the fungus and host have led to the evolution of biochemical pathways for the synthesis of unusual secondary metabolites that have found many potential applications in anticancer and antimicrobial studies (Yarden, 2014; Hasan et al., 2015; Li et al., 2016; Deshmukh et al., 2017). Approximately 21, 19, and 16% of new bioactive metabolites obtained from the marine fungi come from those associated with algae, sponges, and mangrove habitats, respectively (Rateb and Ebel, 2011). Some of these biologically active compounds were products of previously unknown biosynthetic gene clusters identified by sequencing the marine genomes (Kjer et al., 2010; Li et al., 2016; Rédou et al., 2016). However, all existing data from the genome sequencing projects concerned to glycoside hydrolases (GHs) and concomitant enzymes [auxiliary activities (AAs), carbohydrate esterases (CEs)] indicate that marine fungi have developed the metabolic pathways rather related to breakdown of terrestrial plants than algae or animal residues (Arfi et al., 2013; Kumar et al., 2015). Nevertheless, the comparison of the entire repertoires of plant saprophyte metabolic pathways between marine and terrestrial fungi revealed that the terrestrial fungus *Neurospora crassa* has only about half as many protein families linked to sugar uptake (159 vs. 328) compared to the marine fungus *Scopulariopsis brevicaulis*, while both belong to the *Fusarium*/*Nectria* clade. This fact suggests a broadened substrate specificity of the marine fungal enzymes that may be conditioned by the adaptation of once soil fungi to a marine life style in the medium with the higher salt concentrations, depleted nutritional resources and/or fungal-marine habitant relationships (Kumar et al., 2015).

Many proteins encoded by fungal genomes involved in the plant degradation required rather transcriptomic, proteomic or gene functional analyses. These analyses revealed the presence many post-genomic or post-translational modifications during the lignocellulose degradation process, particularly in the presence of salt (Arfi et al., 2013; Panno et al., 2013; Cong et al., 2017). The new multigene transcripts of lignolytic laccases were found in the marine-derived basidiomycete *Peniophora* sp. CBMAI 1063 cultivated in saline conditions (Otero et al., 2017). The presence of salt modified the lignocellulolytic enzyme composition of the salt-adapted mangrove fungus *Pestalotiopsis* sp. NCi6, increasing the number of the secreted GHs that were more diverse (nine vs. six families), and more enriched in cellulolytic AA9 (formerly GH61) and xylanolytic GH43, GH10, and GH30 than in conditions without salt (Arfi et al., 2013).

Thus, the possibility of the secondary colonization of fungi from land to marine ecosystems cannot be excluded. Many unknown fungal species, even at higher taxonomic levels in the *Chytridiomycota* forming an ancient evolutionary lineage, *Ascomycota*, and *Basidiomycota* found in the deep-sea water, and the molecular clock estimates of their rRNA evolution suggested the hypothesis that fungi initially diversified in the ocean before they colonized the land (∼400 million years ago) (Le Calvez et al., 2009; Manohar and Raghukumar, 2013). Moreover, there is abundant evidence for multiple recolonizations of the ocean by fungi (Spatafora et al., 1998; Richards et al., 2012). The genome sequencing of the psychrotrophic strain *Cadophora malorum* revealed deficient in cellulase genes, but its putative alginate lyase could be acquired due to the adaptation to marine environment (Rédou et al., 2016).

If anything, fungi are an important consumer of plant and animal residues as well as chemical pollutions of the marine environments (Harms et al., 2011; Richards et al., 2012). Many extra- and intracellular enzymes of marine fungi such as GHs, nucleases, proteases, and lipases involved in the degradation of cell walls, DNA, proteins, and other organic matter have been structurally or/and biochemically characterized and showed the higher specific activity and effectiveness in comparison with those from their terrestrial counterparts (Nielsen et al., 2007; Kamat et al., 2008; Beena et al., 2011; Harms et al., 2011; Balabanova et al., 2012; van Leeuwen et al., 2012). In addition, marine fungi can produce enzymes with unique specificity toward the marine polymeric substrates such as laminarins, fucoidans, ulvans, carragenans, and agar (Bonugli-Santos et al., 2015; Wang et al., 2016). In particular, the ability to decompose brown algae is the most valuable. It has been found that brown algae evolutionary distinguished from land plants and other algae by their cell wall structure. They contain carbohydrates that are feature of plants (cellulose), animals (fucose-containing sulfated polysaccharides) and bacteria (alginates) (Keeling et al., 2009; Deniaud-Bouet et al., 2017). Therefore, an idea of using marine fungi for the plant and algae biotransformation has been successfully exploited for the production of low-cost edible protein and highly valuable biochemicals, as well as for wastewater treatments (Beena et al., 2011; Harms et al., 2011; van Leeuwen et al., 2012).

Studying the carbohydrate-active enzymes (CAZymes), particularly the main and concomitant polysaccharide-depolymerizing enzymes in marine fungi, allow for the elucidation of mechanisms of their action and advantages for biotechnological use. The comparison of enzyme expression profiles in the dependence on plant or algae polymeric substrates in the growth medium can reveal the nutrition preferences and CAZyme repertoire of the marine fungi. They are potential producers of protein-rich digestible biomass from plant and macroalgae, biotechnology relevant enzymes as well as are new source of drugs and biotechnological discoveries.

## Carbohydrate-Active Enzymes

For the efficient bioconversion of plant and algae material, microorganisms or enzymes capable of degrading the indigestible cell wall polysaccharide complexes are the most valuable for biotechnology. Cellulose, hemicellulose, and pectin are the main polysaccharides of plant cell walls that are strengthened by an aromatic heteropolymer lignin preventing their enzymatic digestion (Ochoa-Villarreal et al., 2012). Cellulose has a linear structure of β-1,4-linked D-glucose residues. The long chains forms microfibrils non-covalently linked together by hemicelluloses. Hemicelluloses are distinguished by the main sugar in the backbone chain: xylan (β-1,4-linked D-xylose), mannan (β-1,4-linked D-mannose) and glucomannans (β-1,3;1,4-D-glucans with mannose), or xyloglucan (β-1,4-D-glucan with β-1,6-attached xylose). The backbone chains of hemicelluloses have many branches as attached monomers of D-galactose, D-xylose, L-arabinose, and D-glucuronic acid. Pectins are differed by three main structures: homogalacturonan (linear polymer), xylogalacturonan (branched by β-1,3-linked D-xylose), and rhamnogalacturonan. Each plant has the different structure and chemical composition of their cell wall layers that is dependent on the species, tissue, and the growth phase (Ochoa-Villarreal et al., 2012).

Fungi have been found to produce a wide range of CAZymes and degrade plant complex polymers into digestible and assimilable products for other members of ecosystems. The CAZymes have been well surveyed in the terrestrial basidiomycetes and ascomycetes (van den Brink and de Vries, 2011; Rytioja et al., 2014). The plant-degrading CAZymes such as cellulases, hemicellulases, ligninases, and pectinases, and the accessory debranching enzymes belong to the following classes: GHs, glycosyl transferases (GTs), polysaccharide lyases (PLs), CEs, and AAs that can be linked to carbohydrate-binding modules CBMs (Guillén et al., 2010; van den Brink and de Vries, 2011; Rytioja et al., 2014). The production of accessory depolymerizing enzymes synergistically working with the backbone-degrading enzymes is regulated mainly at the transcriptional level in filamentous fungi for more deeply degradation of plant polysaccharide complexes (Aro et al., 2005). Nearly 200 CAZyme families with more than 300 representatives have been identified in the predicted fungal proteomes (Zhao et al., 2014). The plant facultative endophytic fungi showed a highest number of CAZymes. It is known that at least 35 GH, 3 CE and 6 PL families are involved in plant polysaccharide degradation (van den Brink and de Vries, 2011). Recently, the lignin-degrading enzymes have been joined to the families LPMO included a new CAZy class of AAs to adopt a range of oxidative mechanisms related to lignocellulose conversion (Levasseur et al., 2013). Comparative analyses of AAs in 41 fungal genomes divided them on several groups and subgroups in dependence on their phylogenetic origin and life style (Hori et al., 2013). Fungi have been found to be the only organisms in which there are all three LPMO families AA9, AA10, and AA11, indicating the importance of the oxidative enzymes promotive of lignocellulose utilization for their lifestyle (Morgenstern et al., 2014).

The genomic or transcriptomic analyses may provide information about the life style and metabolic repertoire of marine fungi. Although the data on marine fungi with the sequenced genomes are restricted, they carry sufficient information about the common ancestral forms of life with terrestrial fungi such as the capability of utilizing plant polysaccharide complexes for their growth (Arfi et al., 2013; Kumar et al., 2015). Moreover, the marine fungi gave a display many additional genes encoding putative CAZymes and their concomitant proteins as compared to the known terrestrial plant-degrading counterparts (Aro et al., 2005; Arfi et al., 2013; Hori et al., 2013; Levasseur et al., 2013; Rytioja et al., 2014; Zhao et al., 2014; Kumar et al., 2015). The genome of the marine strain *S. brevicaulis* LF580 isolated from the inner tissue of a marine sponge has been found to contain the largest numbers of CAZy genes (478), many of which are the putative genes involved into plant polysaccharide degradation: 71 AAs, 34 CEs, 50 CBMs, 227 GHs, 81 GTs, and 15 PLs (Kumar et al., 2015). It contains 21 hydrolases from the GH5, GH6, and GH7 families against only eleven in *Trichoderma reesei*, which is widely used in biotechnology. This suggests that the marine strain *S. brevicaulis* LF580 may be able to degrade a larger variety of plant substrates than some terrestrial lignocellulolytic fungi (Kumar et al., 2015).

However, the regulation of the expression of CAZyme genes at the molecular level have been studied mostly in terrestrial fungi. The genes encoding CAZymes in the presence of polymers or their partially hydrolyzed molecules have been shown to be repressed under the growth conditions on simple substrates such as glucose, when the fungus does not need the production of the polysaccharide-degrading enzymes for the nutrition (Aro et al., 2005). In the same way, low molecular weight carbohydrates produced during destruction of polymers could induce the expression of other CAZyme genes (Coradetti et al., 2012; Hori et al., 2013; Mukherjee et al., 2016). A number of genes encoding cellulases and pectinases in *N. crassa* showed increased levels of the transcripts under carbon starvation and during pretreatment of the culture with cellulose or pectin (Benz et al., 2014). The lignocellulolytic pathways of *Myceliophthora thermophila* varied with different plant substrates, reflecting the plant cell-wall polysaccharide structure and content (Kolbusz et al., 2014). The genes encoding additional xylanolytic enzymes were up-regulated in the presence of monocot straws, while the genes encoding additional pectinolytic enzymes were up-regulated in response to the presence of dicot alfalfa, canola, or flax in the nutrition medium. Analyses of the RNA-Seq data under the cultivation of *Arthrinium malaysianum* with the repressor of glucose uptake 2-deoxy D-glucose (2-DG) revealed that 2691 transcripts were differentially expressed vs. control samples, and 302 CAZyme genes was up-regulated in response to 2-DG (Mukherjee et al., 2016).

Marine fungi also produced enzymatically active cellulases and laccases, or some specific GHs related to the marine origin, when agricultural plant or waste (cotton seed, sugarcane bagasse, rice bran, waste paper, cellulose, sisal waste, molasses spent wash, black liquor, etc.), or algal polysaccharides were added into the growth medium (Raghukumar, 2008; D’Souza-Ticlo et al., 2009; Ravindran et al., 2010; Rodriguez-Jasso et al., 2010; Zhang and Kim, 2010; Chen et al., 2011; Faten and Abeer, 2013; Bonugli-Santos et al., 2015; Hong et al., 2015; Wang et al., 2016; Balabanova et al., 2018). The capability of metabolic utilization of plant or macroalgae polysaccharides allows for an increase in the production of fungal biomass enriched by mycelium proteins and extracellular enzymes that can be used in animal or fish feeding, or in the bioremediation of soils and water (Supplementary Table 1). The unique properties of CAZymes from the marine fungi are important for biotechnology because of their ability to function at the high salinity and pH, low water potential, high sodium ion concentrations, extremely low or high temperature, oligotrophic nutrient conditions, and the high hydrostatic pressure in comparison with the enzymes of terrestrial fungi that are mostly cultivated at pH 4.5–6.0 and low salinity (≤0.05%) (Raghukumar, 2008; Farinas et al., 2010; Pang et al., 2011; Zilly et al., 2011; Arfi et al., 2013; Del-Cid et al., 2014; Lee et al., 2015; Thirunavukkarasu et al., 2015; Dos Santos et al., 2016; Trincone, 2018).

## Enzymes Modifying Macroalgae Polysaccharides

Macroalgae may contain plant-specific cellulose and xylan as well as a range of unusual polymers for land organism such as alginates, fucans/fucoidans, laminarins (brown algae), agar/agarose, carrageenan (red algae), and ulvan (green algae), many of which are sulfated and include monomers of fucose and uronic acids. Thus, algal polysaccharides are more diverse that require additional catalytic mechanisms or metabolic pathways to their fermentation (Vera et al., 2011; Abdallah et al., 2016; Garcia-Vaquero et al., 2017; Trincone, 2018).

**FIGURE 1:**
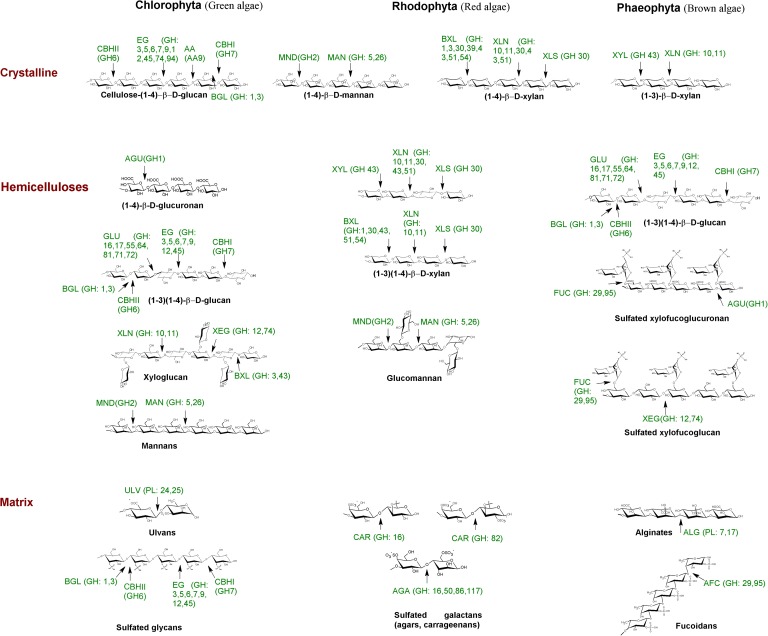
Schematic representation of algae cell wall polysaccharides and corresponding polysaccharide-degrading enzymes. AA, monooxygenase; AFC, α-fucosidase; AGA, agarase; AGU, gucuronidase; ALG, alginate lyase; BGL, β-1,4-glucosidase; BXL, β-1,4-xylosidase; CAR, carrageenase; CBHI, exo-β-glucanase (reducing end); CBHIl, exo-β-glucanase cellobiohydrolase (non-reducing end); EG, endo-β-1,4-glucanase; GLU, β-1,4/1,3-glucanase; MAN, β-1,4-endomannanase; MND- β-1,4-mannosidase; ULV, ulvanlyase; XEG, xyloglucan-β-1,4-endoglucanase; XLN, β-1,4/1,3-endoxylanase; XLS, β-1,4-xylosidase (reducing end); XYL, β-1,3-xylosidase (van den Brink and de Vries, 2011; Pluvinage et al., 2013; Rytioja et al., 2014; Zhao et al., 2014; Zhu et al., 2016; Gao et al., 2017; Ulaganathan et al., 2017). Algae polysaccharides content and structures were presented with the use of data reported by Synytsya et al. (2015). Enzyme abbreviations were applied according the EC and CAZy classification. Algae polysaccharide-degrading enzymes were shortened according to the first letters of the enzymatic names.

Macroalgae polysaccharides are divided into storage and structural depending on their chemical structure and function (Jiao et al., 2011; Kim, 2011; Ermakova et al., 2015; Rodrigues et al., 2015; Synytsya et al., 2015; Abdallah et al., 2016; Cunha and Grenha, 2016; Deniaud-Bouet et al., 2017; Raimundo et al., 2017). Starch is the storage polysaccharide in the green algae chloroplasts similarly to plants (Kim, 2011). The cell walls of green seaweeds are formed by ulvans consisting of sulfated rhamnose residues as the main units linked to uronic acids (Kim, 2011; Vera et al., 2011; Synytsya et al., 2015). They also contain xylan, mannan, and cellulose (**Table 1**). Floridean starch granules outside of plastids, and consisting mostly of a-D-glucose and insoluble amylopectin, are the main storage polysaccharide in the red seaweeds (Kim, 2011). The cell walls of marine red algae have a complex texture due to the content of cellulose, xylan, or mannan fibrils and matrix polysaccharides, including the economically important sulfated galactans such as carrageenan and agar used for the bioethanol production (**Table 1** and **Figure 1**). However, many of them need to be enzymatically pretreated before their use (Synytsya et al., 2015; Abdallah et al., 2016; Trincone, 2018). The main storage polysaccharide in the brown seaweeds is laminarin formed by 1,3-β-glucans with β-1,6-branching and different reducing endings with mannitol or glucose residues (**Table 1** and **Figure 1**). The chemical composition and content of seaweed polysaccharides changes depending on the seasons, age, species, and location (Kim, 2011). The cell walls of brown seaweeds contain mainly fucoidans from different amount of saccharide unites with different degrees of sulfation (Vera et al., 2011; Ermakova et al., 2015; Deniaud-Bouet et al., 2017; Garcia-Vaquero et al., 2017; Trincone, 2018). These polysaccharides consist of α–1,3-backbone or repeating disaccharide units of α–1,3- and α–1,4-bound fucose residues branching at the C2 positions and sulfated at the C4 and/or C2 positions. Additionally, fucoidans may have the mannose, xylose, galactose, rhamnose and uronic acid residues (Kim, 2011; Ermakova et al., 2015; Synytsya et al., 2015). According to the chemical composition of the branches, fucoidans can be divided into xylofucoglycuronans and glycuronogalactofucans (Kim, 2011). Brown seaweeds were reported to contain about 14% of extra carbohydrates in the form of alginate associated with phenolic compounds (Synytsya et al., 2015; Deniaud-Bouet et al., 2017; Raimundo et al., 2017). Alginates are linear polymers composed by two epimers, β-1,4-D-mannuronate (M) and α-1,4-L-guluronate (G) (Synytsya et al., 2015; Deniaud-Bouet et al., 2017). Alginates and fucoidans of brown macroalgae were also required additional enzymatic treatment and saccharification during their conversion into biofuels (Kim, 2011; Abdallah et al., 2016; Trincone, 2018).

**Table 1:** The composition of polysaccharides of food macroalgae fibers.

Macroalgae	Soluble fibers	Insoluble fibers
**Brown algae** (*Phaeophyta*)	**Laminarins:**β-(1,3)-and β-(1,6)-glucose (3:1), mannitol	**Cellulose**β-(1,4)-glucose
*Fucus*	**Alginates:**β-(1,4)-manuronic and α-(1,4)-guluronic acid	
*Laminaria*	**Fucans/fucoidans:**α-L-fucose sulfate, mannuronic acid, xylose, galactose, glucose, mannose	
*Undaria*		
*Himanthalia*		
**Red algae** (*Rhodophyta*)	**Agarose:** D-galactose acid and (3,6)-anhydro-D-galactose sulfate	**Cellulose**β-(1,4)-glucose
*Chondrus*	**Carrageenan:**α-1,3-D-galactose acid and (1,4,3,6)-anhydro-L-galactose sulfate	
*Porphyra*	**Xylan:**β-(1,4)-D-xylose	
*Mastocarpus*	**Mannan:** Mannose	
**Green algae** (*Chlorophyta*)	**Ulvans**: sulfated rhamnose, xylose, uronic acids, glucose	**Cellulose**β-(1,4)-glucose
*Ulva*	**Xylans:** β-(1,4)-D-xylose, arabinose, glucose, galactose, uronic acids, mannose	
*Enteromorpha*	**Mannan:** Mannose **Sulfated galactans**	


The fungal enzymes degrading algal polysaccharides can be categorized into the same protein families and classes according to the CAZy classification as the plant-polysaccharide-degrading enzymes (**Figure 1** and Supplementary Table 2). The β-1,3-linkage, which is abundant in marine substrates, has been found to be degraded by enzymes belonging to the GH families: GH3, GH5, GH16, GH17, GH26, GH55, GH64, GH81, and GH131 (**Figure 1** and Supplementary Table 2b). Cleaving β-1,3-linkage by these GHs might occur in concert with auxiliary domains for their action against recalcitrant substrates (Guillén et al., 2010). In the cases of putative cleaving β-1,3-glucans, the auxiliary domains CBM43 and CBM13 were shown to associate with GH5 and GH17, respectively (Blackman et al., 2014). Family 6 CBMs are appropriate receptors for laminarin due to the presence of multiple distinct ligand binding sites (van Bueren et al., 2005). An enzyme with GH5 and GH26 catalytic domains that possessed beta-1,3-1,4-endoglucanase activity contained CBM11 (Carvalho et al., 2004). The degrading activities toward β-1,6-bonds remain poorly known and are found in GH5, -13, -30 of marine origins, and in a new GH131 family of fungal proteins (Supplementary Table 2b). However, there are enzymes with unique structures and specificities related to the substrates of marine origin such as the recently determined fucoidanases of the GH107 family, α-agarases of the GH117 family, or ulvan lyases of PL24 and PL25 families predominantly occurred in marine bacteria (Supplementary Table 2b and **Figure 1**). Unfortunately, marine fungal enzymes specific toward to the algal polysaccharides have yet to be structurally determined and classified (Supplementary Table 1).

Thus, an alginate lyase from the *Aspergillus oryzae* associated with brown seaweed was unique due to cleaving the β-1,4 glycosidic bond between polyM and polyG blocks of sodium alginate resulted in a higher polyM/polyG ratio in comparison with the acid hydrolysis (Singh et al., 2011). The marine isolates *Calcarisporium* sp. KF525, *S. brevicaulis* LF580, and *Tritirachium* sp. LF562 as well as isolates from other experiment, *A. oryzae* and *Dendryphiella salina*, produced biomass from alginate (Moen et al., 1995; Singh et al., 2011; Wang et al., 2016).

Only *Calcarisporium* sp. KF525 could additionally produce biomass from the sulfated galactans, agar and carrageenan (Supplementary Table 1). Among 18 marine-derived morphospecies, *Phoma* sp., *Aspergillus ochraceus*, and *A. terreus*, possessed also carrageenase activity (Solis et al., 2010). However, all 144 studied fungal isolates (except *Fusarium* sp.) grew with carrageenan as the sole carbon source, 10 of which produced the largest mycelial biomass. Several CAZymes from the families GH11, -82, and GH50, -86, -117, -118 found in bacteria are known to contain activities related to carrageenase and agarase, respectively (**Figure 1** and Supplementary Table 2b). Carrageenases and agarases have not yet been explored in marine fungi, but these enzymes can belong to the multifunctional family GH16, whose genes are widely distributed in fungal genomes (Zhao et al., 2014; Kumar et al., 2015; Mai et al., 2016) (Supplementary Table 2b).

Some marine fungal strains grew on the ulvan-containing material, indicating that they may be a source for novel ulvan lyases and GHs as it was found in marine bacteria producing the enzymes of new families PL24-25 and GH105 (Solis et al., 2010; Collén et al., 2014; Gnavi et al., 2017; Ulaganathan et al., 2017). The fungal endo-β-1,4-glucuronan polysaccharide lyase isolated from *T. reesei* was applied to the glucuronan depolymerization of the green seaweed *Ulva lactuca* for the production of bioactive glucuronic acid oligosaccharides (Redouan et al., 2009). The enzyme crystal structure was determined at 1.8 Å resolution as the first three-dimensional structure of the PL20 family (Konno et al., 2009).

Cultivating the marine fungi on the different plant and algal substrates induced also the production of proteases, amylases, glucanases, xylanase, pectinases, and lipases (Nadu et al., 2011; De Souza et al., 2015; Wang et al., 2016; Balabanova et al., 2018). The utilization of laminarin, starch, and xylan by *Calcarisporium* sp. KF525, *Tritirachium* sp. LF562, *Bartalinia robillardoides* LF550, *Penicillium pinophilum* LF458, *S. brevicaulis* LF580, and *Pestalotiopsis* sp. KF079 at a similar rate as glucose demonstrated the efficiency with which their amylases and glucanases were expressed (Wang et al., 2016) (Supplementary Table 1). Among the extracellular enzymes of 90 marine fungal strains degrading polysaccharides, amylases and β-1,3-glucanases were most widespread, particularly in the genera *Fusarium, Geomyces*, and *Echinobotrium*, whereas the enzymes cleaving CM-cellulose, agar, and fucoidan were rare (Burtseva et al., 2003). Although the marine fungi *Isaria felina* (accepted name *Beauveria felina*) and *S. brevicaulis* grew well on the submerged rice bran without the addition of salts, they exhibited mainly the β-1,3-glucanase and polymannuronate lyase activities reached for 7 days to 600 U/mg and 280 U/mg, respectively (Balabanova et al., 2018). However, their enzymatic profile included about 25–37 U/mg activities of agarase, alginate lyase, carragenase and fucoidanase during the first 4 days of cultivation dropped almost to zero at the following 7 days, probably, due to the growth on the plant substrate (Balabanova et al., 2018). Twenty-four GH3, 13 GH5, 11 GH13, 12 GH16, 4 GH17, 5 GH55 that can relate to β-1,3;1,6-glucanase activity have been found in the marine *S. brevicaulis* LF580 grown at the highest rate on laminarin as the sole source of carbon (Supplementary Tables 1, 2). β-1,3-Glucanases are the most biochemically studied enzymes in the marine fungi because β-1,3-glucans are polyfunctional important components of the fungal cell wall as well as of many plants and algae (Burtseva et al., 2003; Sova et al., 2013; Raimundo et al., 2017). The fungus *Chaetomium indicum* associated with *Fucus evanescens* collected near the Kuril Islands, and *Trichoderma aureviride* sampled from bottom sediments of South China Sea had similar extracellular laminarinases classified as exo-1,3-β-D-glucan-glucanohydrolases (EC 3.2.1.58): the temperature optimums (40–45°C), molecular masses (54–56 kDa), Km (0.1–0.3 mg mL^-1^) (Burtseva et al., 2003). However, temperature stability of laminarinase of *C. indicum* was significantly higher than the enzyme from *T. aureveride*. Many endo-β-1,3-glucanases and glucan-binding proteins of marine origin have been found to belong to GH16 (Sova et al., 2013). Remarkably, only fungi possessed β-1,3-glucanases with exo-type action. However, their belonging to any GH family has yet to be determined (Sova et al., 2013).

The microbial producers of fucoidanases are rare and the enzyme properties are poor studied despite the biotechnology potential of fucoidans (Ermakova et al., 2015; Trincone, 2018). Although fucoidans constitute up to 25–30% of the seaweed dry weight dependent on the species and season, no more than 20 fucoidanases of the microbial producers have been characterized (Rodriguez-Jasso et al., 2010; Ermakova et al., 2015; Trincone, 2018). It has been shown that while fucose was well consumed by several fungal species, their growth on fucoidan did not allow the biomass production, indicating the absence of fucoidanases in these marine fungi (Wang et al., 2016). In addition, the absence of simple methods for quantitative determination of the fucoidanase activity and the use of structurally uncharacterized fucans hamper exploring fucoidanases and finding the new enzymes (Ermakova et al., 2015). The enzymes distinguishing by structures and consequently by the substrate specificities could be involved in the transformation of fucoidans with unknown diverse structures.

Probably for the same reason, the sequenced marine strain *Scopulariopsis brevicaulis* LF580 growing on alginate or ulvans as the sole carbon source does not have any known families of algae polysaccharide-degrading enzymes such as alginate lyases (PL7,-15,-17) or ulvan lyases (PL24,-25) (Supplementary Tables 1, 2a,b). At the same time, marine fungi associated with macroalgae may be depleted in some enzymatic activities due to their mutualistic living in microbial communities enriched in the bacteria degrading the algae polysaccharides (Pluvinage et al., 2013; Nedashkovskaya et al., 2014, 2018; Kusaykin et al., 2016; Zhu et al., 2016; Gao et al., 2017; Raghukumar, 2017). Thus, only two thermostable (50–60°C) fucoidanases from marine fungi *Dendryphiella arenaria* TM94 and *Fusarium* sp. LD8 have been studied to date (Ermakova et al., 2015). However, brown algae have the highest diversity of fungal endophytes such as facultative marine *Aspergillus*, *Cladosporium*, and *Penicillium*, and obligate marine *Halosigmoidea marina* and *Acremonium fuci*, whose population may increase in dead algae (Raghukumar, 2017). Bacteria have been suggested to play a more important role in the submerged macroalgae degradation than fungi (Raghukumar, 2017).

## Amylolytic Enzymes

In fungi, three types of amylolytic enzymes are produced: α-amylase (EC 3.2.1.1), glucoamylase (EC 3.2.1.3) and α-glucosidase (EC 3.2.1.20) belonging to the GH13, GH15 and GH31 families (Chen et al., 2012). Amylases classified as α-1,4- and 1,6-glucanases randomly hydrolyze starch, a storage polysaccharide, to give diverse products such as dextrins and smaller polymers. Polyextremophilic characteristics of α-amylases from marine fungi are often of interest due to their frequent use (25% of total enzyme market) in food, pharmaceutical, and detergent industries (Ali et al., 2014).

An α-amylase from hyper halophile *Engyodontium album* TISTR 3645 purified up to the specific activity 132.17 U mg^-1^ was able to work at a high salinity 30% and temperatures 70–80°C that made it a useful candidate for bioremediation as well as various industries where a higher salt concentration, surfactants, and detergents inhibit enzymatic conversions (Ali et al., 2014). *A. oryzae* and *Penicillium* sp. isolated from marine sediments collected in the east coast of India showed the high levels of amylase activity (220–250 U mg^-1^), whose biomass was grown by solid state fermentation (SSF) with the use of spoiled banana fruit with starch supplementation at 35–40°C and pH 6.5 (Sathya and Ushadevy, 2013). The fungus *Penicillium* sp. NIOM-02 showed an increase of amylase activity at cultivation on wheat and corn flour by submerged fermentation (SmF) (246 U mg^-1^) and by SSF (18 U mg^-1^) in comparison with the activity on sugar cane bagasse (Dhale and Vijay-Raj, 2009). Remarkably, the addition of higher quantities of corn flour or corn steep liquor (≥30 g L^-1^) as the substrate repressed the amylase production during SmF. Moreover, an acidic condition did not stimulate the fungus metabolite production, inducing only stress-dependent sporulation, probably, due to the marine origin of the strain, where pH is slightly alkaline (Dhale and Vijay-Raj, 2009).

## Cellulolytic Enzymes

It is known that the enzymatic breakdown of cellulose in fungi is achieved by GHs from the families 5, 6, 7, 12, and 45 distinguished by the mode of enzymatic action and the substrate specificity: cellulose 1,4-β-cellobiohydrolyses (reducing end) (EC 3.2.1.176; CBH I; GH7); β-1,4-endoglucanases (EC 3.2.1.4; EG; GH 5,6,7,12,45), exo-β-glucanases or cellobiohydrolases (non-reducing end) (EC 3.2.1.91; CBHII; GH 6,7), β-glucosidases (EC 3.2.1.21; BGL; GH 1,3), and the auxiliary enzymes (AA) (Supplementary Table 2). Endoglucanases catalyze the cleavage of accessible intramolecular β-1,4-glucosidic linkages in cellulose randomly and production of the new chain ends (Payne et al., 2015). Exoglucanases processively hydrolyze cellulose chains at the ends up to soluble cellobiose or glucose, and then β-glucosidases cleave cellobiose to glucose, eliminating cellobiose-dependent inhibition. Oxidative enzymes are in 12 AA families, of which 8 AAs act during lignin degradation and 4 AAs act on polysaccharides (LPMOs) with an endo-type mechanism of action in crystalline regions of the chains (Payne et al., 2015). The present microbial cellulase production technologies including genetic optimization of the strains have reached an industrial level of research (Ochoa-Villarreal et al., 2012; Rytioja et al., 2014; Payne et al., 2015; Kuhad et al., 2016). The cellulase complex of fungal species of genera *Trichoderma* and *Aspergillus* are believed to be the most equipped for plant material degradation and therefore their genetic-engineering strains, particularly *T. reesei*, and the genes encoding highly active cellulases, have been intensively used for the improvement of industrial processes (Payne et al., 2015; Kuhad et al., 2016; Druzhinina and Kubicek, 2017).

Although cellulose is a major component of the terrestrial plant material, many marine fungi also possess cellulase activity and are able to grow well on pure cellulose as well as on plant wastes (Ravindran et al., 2010; Solis et al., 2010; Borines et al., 2011; Nadu et al., 2011; Faten and Abeer, 2013; Sathya and Ushadevy, 2013; Ali et al., 2014; Alsheikh-Hussain et al., 2014; Bonugli-Santos et al., 2015; Hong et al., 2015; Balabanova et al., 2018). About 3.5–4.6% and 11.5–16.1% of cellulose fiber were chemically determined in non-food macroalgae *Ascophyllum nodosum* and *Sargassum* sp., which were used for bioethanol production (Kraan, 2012). The cellulose and hemicellulose content of the seaweeds has been surveyed to be 2–10% and 9% dry weight, respectively. Cellulose was one of the most preferred carbon source for nine fungal strains among 18 marine-derived species (144 strains) in the study of Solis et al. (2010). However, none of six marine strains reported by Wang et al. (2016), *Calcarisporium* sp., *Tritirachium* sp., *Bartalinia robillardoides*, *Penicillium pinophilum*, *Scopulariopsis brevicaulis*, and *Pestalotiopsis* sp., grew well on cellulose or CMC as the sole carbon source indicating only weak production of cellulases or endoglucanases. The initial cellulase activity of the marine strains *B. felina* and *S. brevicaulis* grown on the rice floury bran reached to 27 and 45 U/mg, respectively (Balabanova et al., 2018). However, the value increased approximately threefold after the week of cultivation in only *S. brevicaulis*. An effective cellulase production has been reported by the marine fungus *Cladosporium sphaerospermum* isolated from deteriorated seaweed *Ulva* through SSF and its potential in the saccharification of the green seaweed *U. fasciata* for bioethanol production (Trivedi et al., 2015). Cellulase-producing marine fungi among 181 samples isolated from the continental slope sediments of the Arabian Sea belonged mainly to genera *Cephalosporium* (36.5%), *Pleospora* (22.5%), *Humicola* (20.5%), and *Penicillium* (18.55%) (Smitha et al., 2014). Thus, not all marine strains of fungi are producers of active cellulolytic enzymes; probably, due to their specialization to the more frequently occurred substrates in the marine environment than cellulose. Facultative marine strains related to the plant cell wall degradation are more likely to be cellulolytic (Supplementary Table 1).

However, the use of cellulose in the growth medium as the sole carbon source can provide an increase of cellulolytic enzymes synthesis in fungi (Hong et al., 2015; Lee et al., 2015). An endophyte *Arthrinium malaysianum* closely related to *A. arundinis, A. saccharicola* from marine brown algae *Sargassum* sp. has the innate ability to produce extracellular lignocellulose-degrading enzymes (Hong et al., 2015; Mukherjee et al., 2016). The higher levels of the expression of extracellular endoglucanase (EG), β-glucosidase (BGL), β-xylosidase (BXL), filter paper activity (FPase) under the 2-deoxy D-glucose (2-DG) treatment were exploited for evidence of the enzyme genes up-regulation (Mukherjee et al., 2016). The exogenous addition of 2-DG to fungal cells in a growth media caused the glucose starvation-like response. The results showed that 16 β-glucosidases of the GH1, and 6 glucan β-1,3-glucosidases of the GH5 family involved in cell wall biogenesis/degradation was significantly up-regulated. Moreover, *in vitro* addition of the non-metabolized glucose analog 2-DG in the medium containing cellobiose resulted in a further increase of the β-glucosidase and endoglucanase activities. The presence of numerous cellulose- and xylan-degrading enzymes of the GH5, GH3, GH16, GH43 families allow considering the strains of *Arthrinium* spp. as an important candidates for biofuel production (Mukherjee et al., 2016). The enzymes assay including the determination of filter paper units (FPU) related to saccharification yield; EG activity attacking the non-crystalline cellulose, and BGL activity promoting the cellulase inducers expression, revealed the highest values of the cellulolytic activity in the marine fungus *A. saccharicola* (Supplementary Table 1).

The activities of marine fungi cultured in non-marine media were comparable to the reported values of the terrestrial wood-decaying fungi (Hong et al., 2015). In a previous study, the total cellulase activities for several strains isolated from a coastal marine sponge *Haliclona simulans* were similar to the activities for the cellulase-overproducing mutant *Hypocrea jecorina* QM9414 (*T. reesei*) on all types of saline and non-saline media (Baker et al., 2010; Hong et al., 2015) (Supplementary Table 1). The marine-derived strains Basidiomycete MEG2, Pezizomycetes CMCA22 and GPG3 showed an increased the EG activity at low temperatures with the addition of sophorose precursor, cellobiose (Alsheikh-Hussain et al., 2014).

The majority of 18 marine-derived ascomycetes and zygomycetes also showed the EG and BGL activities independently on salinity (Lee et al., 2015). Only the cellulolytic activity of *Penicillium chrysogenum* increased with a salinity 0.5 M at pH 7–8 that corresponds to the values of the ocean. *Arthrinium phaeospermum* and *Fusarium equiseti* grew with the highest rate in saline conditions, indicating the intrinsic halo-tolerance due to the long-time adaptation to a marine life style (Lee et al., 2015). However, mangrove fungus *Pestalotiopsis* sp. NCi6 capable of utilizing complex lignocellulosic substrates in the presence of high concentrations of salt was distinguished by lignocellulolytic profiles of the secretomes in non-saline and saline conditions (Arfi et al., 2013) (Supplementary Table 2). Although the *Pestalotiopsis* sp. NCi6 transcriptome was more enriched in lignin breakdown enzymes, the proteomic and transcriptomic analyses suggested that the adaptation of mangrove fungi to salt expressed in an increase in the number of cellulolytic enzymes, enhancing cellulose and hemicellulose hydrolysis at increasing the salinity up to 3% (Arfi et al., 2013).

Considering the importance of cellulases with the alkaline pH-optimums in craft pulping industries, screening of the marine-derived endophytes and wood litter fungi has been carried out in the mangrove ecosystem of the Goa coast using agro-wastes (Ravindran et al., 2010). Among 54 strains, *Aspergillus* sp. and *Chaetomium* sp. isolated from wood litter showed a higher level of exoglucanase (FPase), EG and BGL activities at pH 9.7 grown on cottonseed as the carbon source. The other fungal strains demonstrated average, weak or no activity of the enzymes. Cellulolytic activity was also high in the marine fungus *Helicascus kanaloanus* associated with the Indian mangrove driftwood samples (Nadu et al., 2011).

Although the strain *S. brevicaulis* LF580 was isolated from the inner tissue of the marine sponge, it was fully equipped with putative enzymes involved in cellulose degradation similarly to other ascomycetes able to modify or deconstruct plant material (Supplementary Tables 1, 2). The plant polysaccharide-degrading enzymes were also predominant in *Pestalotiopsis* sp. NCi6 in comparison with the amount of putative algae polysaccharide-degrading enzymes (Supplementary Table 2).

## Hemicellulose-Degrading Enzymes

Hemicellulose polymers consist of pentoses (xylose and arabinose), hexoses (mostly mannose), and a number of sugars and acids. Consequently, several enzymes are needed to completely degrade these polysaccharides. The hemicellulases include endo/exo xylanases, endo/exo-β-glucanases, β-mannanases, arabinofuranosidases, and feruloyl esterases, acting on specific glyco-units and glycosidic bonds toward different hemicelluloses (Supplementary Table 2). Xylan is the major component of hemicellulose in the plant cell wall. Various forms of xylanases exist in nature, which belong to the GH families 1, 3, 10, 11, 30, 39, 43, 51 with the predominance of GHs 10, 11 and 30 in fungi. β-Xylosidases are grouped into the GH families 3, 8, 30, 39, 43, 52, 54, 116, 120, but the known GHs of fungal origin are limited to families 3 and 43 (Ochoa-Villarreal et al., 2012; Rytioja et al., 2014; Kirikyali and Connerton, 2015; Berlemont, 2017; Thomas et al., 2017). Xylanases are used concurrently with cellulases and pectinases for clarifying juices, the liquefaction of vegetables and fruits as well as in the pretreatment of forage crops to improve the digestibility of ruminant feeds and to facilitate composting (Nadu et al., 2011; Goddard-Borger et al., 2012).

Xylans of different chemical structures forming a backbone with β-1,3-xylopyranosyl linkages are only found in marine macroalgae (Goddard-Borger et al., 2012; Synytsya et al., 2015) (**Figure 1**). In some macroalgae, where cellulose is absent (*Chlorophyceae* and *Rhodophyta*), xylan forms a highly crystalline fiber-like material. The main polysaccharide in *Acetabularia*, *Codium*, and the *Halicoryne* genera, and in some red algae such as *Porphyra umbilicales* is β-1,4-mannan, which is a structural and reserve component of green algae (siphonaceous) (Goddard-Borger et al., 2012; Synytsya et al., 2015) (**Figure 1**).

Since some seagrasses and macroalgae showed up to 40% xylan (in red/green algae and higher plants) or fuco-glucuronoxylans (in brown algae) of the polysaccharide content, it was suggested the marine bacteria and fungi associated with them could evolve the efficient mechanisms for xylan degradation at the genetic and/or molecular levels (Kraan, 2012; Del-Cid et al., 2014; Dos Santos et al., 2016). However, the global significance of mycobionts of seagrasses, particularly associated with the roots of aquatic plants, is not well understood (Kohout et al., 2012; Vohník et al., 2016).

Filamentous fungi were found to be one of the best degraders due to their great capability of secreting a wide range of xylan-degrading enzymes that have great biotechnological potential in the paper, pulp, feed, and food industries as well as in the generation of liquid fuels and chemicals from lignocellulose (Lio and Wang, 2012; Dos Santos et al., 2016; Berlemont, 2017).

Algal endophytes *Trichoderma harzianum* and a marine-derived fungus *Aspergillus cf. tubingensis* LAMAI 31 have been found to be effective sources of the highly active salt-inducible xylanases, utilizing xylan as well as agro-industrial residues such as sugar cane bagasse, wheat bran, and rice straw (Thirunavukkarasu et al., 2015; Dos Santos et al., 2016).

Among 493 marine-derived fungi studied by Dos Santos et al. (2016), 112 isolates were able to degrade xylan. Notably, the largest number of fungi able to produce xylanase with an enzymatic activity from 0.25 to 49.41 U mL^-1^ was recovered from marine sponges.

*Cladosporium* sp. associated with Antarctic marine sponges showed the higher xylanolytic activity at low temperatures when grown on beechwood or birchwood xylan and wheat bran, than on wheat straw and oat bran (Del-Cid et al., 2014) (Supplementary Table 1).

The cold-active xylanases from psychrotrophic marine fungi were successfully cloned and expressed in *Escherichia coli* and *Pichia pastoris*. The xylanase gene product with a sequence corresponding to the GH 10 family of the cold-adaptive *P. chrysogenum* FS010 isolated from deep-sea sediments of Yellow Sea was synthesized with the use of an expression vector pGEX-4T-1 (Hou et al., 2006). The higher xylanase activity was registered at the enzymatic production of reducing-sugar ends from birchwood xylan, oat spelts xylan, and wheat arabinoxylan (Supplementary Table 1).

Mangrove fungi producing thermostable and active xylanases in the presence of residual sulfated lignin are highly desirable in the enzymatic treatment of wood pulp after alkaline extraction. The cellulase-free filtrate of *Aspergillus niger* isolated from the detritus of decaying mangrove leaves grown on oat spelts xylan or sugarcane bagasse containing 580 U L^-1^ of xylanase could bleach sugarcane bagasse pulp for 60-min at 55°C (Raghukumar et al., 2004a). The fungal filtrate also showed moderate activities of xylosidase (0.26 U mL^-1^) and arabinofuranosidase that could act synergistically with xylanase at attacking xylan. After purification of the *A. niger* xylanase, its activity reached the value 2457 U mg^-1^ protein, which was comparable with the terrestrial analog (Supplementary Table 1).

Low hemicellulase activities in the marine-derived fungi from brown algae [≤0.02 U of β-xylosidase (BXL) activity per mL of crude fungal extract] were suggested to be due to the low content of hemicellulose in brown algae (Borines et al., 2011; Hong et al., 2015) (Supplementary Table 1).

The high levels of mannanase activity comparable to the cellulase and xylanase activities were determined in all 11 marine fungal strains studied by Arfi et al. (2013) except for two isolates that showed overall low mannanase activity or low activity in the non-saline medium due to an adaptation of certain enzymes to various levels of salinity (Supplementary Table 1). An effective role of mannanases is in the bleaching process to reduce the environmentally harmful chemicals in pulp and paper industry (Arfi et al., 2013).

## Pectinolytic Enzymes

Pectin a heteropolysaccharide composed of α-1,4-linked galacturonate chains with a high percentage of methyl esterification is found in the middle lamella of the plant cell wall and important for controlling growth, wall porosity, and regulation of the ionic environment in plant cells (Eder and Lütz-Meindl, 2008). There are at least nine families of the pectin-specific enzymes, including GH28, GH53, and GH93, polysaccharide lyases of PL1, PL3, PL4, and PL11, and CEs of CE8 and CE13. Enzymes of GH28 are important for the degradation of pectin backbones by fungi (van den Brink and de Vries, 2011). Pectinases such as polygalacturonase (PG) and pectate lyase (PL) are the first enzymes to be secreted by fungal pathogens when they attack plant cell walls (Niturea et al., 2008). The facultative fungal endophyte *Fusarium moniliforme* isolated from decaying leaves of mangrove plants in the saline detritus-rich mud of a mangrove estuary on the west coast of India was a highly pectinolytic producer (Niturea et al., 2008). The fungus was also able to grow without salt and produced maximum biomass and pectinolytic enzymes (PG I, PL) in a liquid medium (Supplementary Table 1). The salt concentration up to 0.4 M NaCl slightly decreased their production, suggesting that although it had been isolated from a halophytic environment, it was not an obligate fungus (Niturea et al., 2008). However, the pectin-depolymerase activities were often not found in some marine fungi, possibly due to the structure-function features of the enzymes, or the absence of their up-regulation in the presence of plant-derived substrates used in experiments. A high occurrence of polygalacturonase producers (30%) among the deep-sea yeast collected from the mud of Sagami Bay (1100–1400 m) capable of degrading plant pectin was inexplicable (Minegishi et al., 2006). Yeasts lost almost all of their pectinases as they adopted to consume simple sugars (Chang et al., 2015). The authors related the capability of utilizing plant pectin non-preferable for yeast to highly reversible metabolic pathways of the deep-sea habitants living in the conditions of nutrient depletion for their growth (Minegishi et al., 2006).

Pectic polysaccharides and the genes for their synthesis have only been identified in the land plants and in the allied streptophyte algae (Chang et al., 2015). Among 103 fungal genomes examined, 21 lacked any PL genes (Zhao et al., 2014). Phylogenetic analyses of fungi indicated that the earliest fungi had copies of GH28 genes, which further duplicated during the evolution of the common ancestor of Chytridiomycota and the terrestrial fungi to be able to consume nutrients from pectin-containing streptophyte plants (Chang et al., 2015). The fungal pectinolytic enzymes that degrade multiple pectic molecules have been suggested to be good indicators of the association between fungi and the land plant lineage (Chang et al., 2015). The pectin-like structures in macroalgae may be different from those of the higher plants through the higher galacturonic and glucuronic acid content as well as an uncommon glucuronic acid-galactose disaccharide (Eder and Lütz-Meindl, 2008, 2010). These structural variations led to the differences in the antibody binding of pectic epitopes in algae and higher plants. A different polysaccharide structure as well as cell wall properties and functions of the unicellular green algae could explain a reduced activity in its pectin methylesterases (PME) in comparison to the higher plants (Eder and Lütz-Meindl, 2008, 2010). Therefore, marine fungi can also possess pectinolytic enzymes with unusual structure and specificity distinguished by the affinity to the algal pectin-like polysaccharides.

## Lignin- and Tannin-Degrading Enzymes

Ligninolytic enzymes play a crucial role in carbon recycling. One of the most important use of these enzymes is in bioremediation to degrade or neutralize pollutants in the environment or to decolorize dyes in industries (Raghukumar, 2008; Sette and Santos, 2013). Taking into account that environmental pollution is largely related to the saline conditions, the use of the lignin-degrading enzymes from the marine-derived fungi can be considered strategic (Zilly et al., 2011; Sette and Santos, 2013; Bovio et al., 2017; Barnes et al., 2018). Lignocellulolytic fungi are classified according to the step of the plant degradation: soft-rot, brown-rot, and white-rot fungi (Hori et al., 2013; Levasseur et al., 2014). A recognized model system for the study of the enzyme machinery involved in the complete degradation of lignocellulosic material is the white-rot fungus *Pycnoporus cinnabarinus*, whose genome contains a versatile ligninocellulolytic enzymatic spectrum (Levasseur et al., 2014). Among the enzymes involved in lignin degradation, *P. cinnabarinus* is known to produce a high-redox-potential laccase of the AA1 family up to 1 g per liter (Levasseur et al., 2014). In addition, white-rot fungi have up to 12 members of ligninolytic peroxidases from the AA2 family, distinguishing them from brown-rot fungi, which contain no AA2 members (Floudas et al., 2012; Hori et al., 2013; Levasseur et al., 2013).

The potential of many lignin-degrading marine fungi lies in their laccases that have been applied for bioconversion of agriculture plants and their wastes in valuable products such as feed supplementations or pharmaceuticals; for biobleaching of paper pulp, dye bleaching in textile industries, wastewater treatment, removing of phenolic compounds in beverages, and biofuel production (Raghukumar et al., 2004b; Raghukumar, 2008; D’Souza-Ticlo et al., 2009; Chen et al., 2011; Feng et al., 2013; Sette and Santos, 2013; Bonugli-Santos et al., 2015). Laccase a multicopper oxidase (benzenediol:oxygen oxidoreductases, EC 1.10.3.2) reduces oxygen to water and simultaneously carries out one-electron oxidation of aromatic, mainly phenolic compounds because of their low redox potentials from 0.5 to 1.0 V to allow for electron abstraction by the Cu1 (Giardina et al., 2010; Desai and Nityanand, 2011). However, some low-molecular intermediate substrates (redox mediators) allow laccases indirectly oxidize large molecules with a high redox potential, including non-phenolic lignin. In the oxidative method for the industrial decolorization or delignification with the use of laccase or the laccase/mediator system, the redox potential difference between the enzyme and the substrate is a relevant indicator of its biodegradability (Giardina et al., 2010; Desai and Nityanand, 2011). Lignin peroxidase (LiP) (E.C:1.11.1.14) and manganese-dependent peroxidase (MnP) (EC 1.11.1.13) and laccase (Lac) (EC1.10.3.2) are the other major lignin-degrading enzymes with great potential for industrial applications (Bonugli-Santos et al., 2010, 2015; Panno et al., 2013). LiP is a heme protein with a high oxidation potential to be able to oxidize phenolic and non-phenolic substrates. MnP is a glycoprotein dependent on H_2_O_2_ and Mn^2+^ and oxidizes aromatic phenols and dyes (Bonugli-Santos et al., 2010).

Lignin-degrading marine fungi have been mostly identified in mangroves and seagrasses (Raghukumar, 2008; Arfi et al., 2013; Panno et al., 2013; Sette and Santos, 2013; Bonugli-Santos et al., 2015). Although mangrove fungi are adapted to high salinity, seawater can influence their growth and enzyme production, suggesting a mechanism of regulation at the mRNA level under hypersaline conditions. The marine strains of the white soft-rot fungi *Pestalotiopsis* sp. NCi6 and *Phlebia* sp. MG-60 have been found to synthesize new transcripts of lignolytic enzymes (isozymes) in secretomes produced in saline conditions (Kamei et al., 2008; Arfi et al., 2013). The laccase activity was completely inhibited, and the number and diversity of ligninolytic enzymes decreased in *Pestalotiopsis* sp. NCi6 in the presence of salt, but simultaneously with an increase of xylanase and cellulase activities (Arfi et al., 2013) (Supplementary Table 2). The expression of two additional isozymes of the lignolytic manganese peroxidases (MnP) in *Phlebia* sp. MG-60 was stimulated in nitrogen-limited medium containing 3% (wt/vol) sea salts that increased the total MnP activity compared to the activity in a non-saline medium (Kamei et al., 2008).

The most represented genera *Penicillium, Cladosporium*, and *Acremonium* associated with seagrass, *Posidonia oceanica*, were rich in the strains able to produce ligninolytic enzymes and tannases useful at degrading and detoxifying lignocellulose residues in the presence of high salt concentrations (Panno et al., 2013). The expression of oxidative enzymes was monitored through decoloration of the dyes Remazol Brilliant Blue (RBBR) for laccases and Amaranth Red (AmR) for peroxidases with the redox potential similar to the natural substrates of these enzymes (Desai and Nityanand, 2011; Panno et al., 2013; Bonugli-Santos et al., 2015). The ascomycetes *Cladosporium cucumerinum* MUT 4296, *Pleosporales* sp. MUT 4399, and white-rot fungus *Schizophyllum commune* (KC339233) showed the high levels of laccase and peroxidase activities (degradation > 75%, DP), respectively (Panno et al., 2013). Approximately 50% of 88 the tested strains were able to decolorize the dyes extensively (DP > 50%) at concentrations 15 and 30 g/l of salts. Only two strains *Beauveria bassiana* MUT 4288 and *Myrothecium roridum* MUT 4326 decolorized the dyes exclusively in the absence of salt, indicating their non-marine origin (Panno et al., 2013). The true marine fungi are active in such extreme conditions as the presence of high salt concentrations that trigger or increase the expression of specific enzymes. Thus, the lignicolous marine fungus *Havispora longyearbyenensis* from Arctic water significantly reduced even the growth without salinity at 4°C (Pang et al., 2011). The halotolerant enzymes from marine fungi may be of great interest in industries where NaCl inhibits their terrestrial counterparts from basidiomycetes, for example in textile wastewaters (Zilly et al., 2011).

Remarkably, there are rather less data about marine ligninolytic basidiomycetes. Three strains *Marasmiellus* sp. CBMAI 1602, *Peniophora* sp. CBMAI 1063, and *Tinctoporellus* sp. CBMAI 1601 were isolated from the marine sponges of the north coast of Brazil (Menezes et al., 2010; Otero et al., 2017). An intensely brown spent wash of molasses (MSW) was decolorized by 60–73% by a marine white-rot basidiomycete, *Flavodon flavus*, immobilized on a polyurethane foam, which could be effectively used for a minimum three cycles (Raghukumar et al., 2004b). Aside from decolorization, the fungus removed 68% of the toxicity of MSW containing benzo(a)pyrene, polycyclic aromatic hydrocarbon (PAH), therefore in the estuarine fish, *Oreochromis mossambicus*, it was showed no liver damage in contrast to the fish after contact with untreated MSW by the fungus (Raghukumar et al., 2004b).

Tannins are the second most represented group of plant phenolic compounds linked to the cell wall polysaccharides after lignin (Chamorro et al., 2012). The polyphenolic compounds involved in a defensive function have been found to be extremely abundant in the seagrass, *P. oceanica*, mainly in rhizomes and leaves (Panno et al., 2013). Many of the fungal isolates associated with the seagrass belonged to Ascomycota and were able to produce tannases. Twenty-nine of them showed more than 50% of the tannase activity in the saline conditions that could make the plant eatable to most of the fauna present in the sea (Panno et al., 2013). The acidophilic tannase produced by marine *Aspergillus awamori* BTMFW032 showed an industrial potential for the synthesis of antioxidant propyl gallate by transesterification, tea cream solubilization, and the simultaneous production of tannase and gallic acid (Beena et al., 2011). The medium optimization indicated that productivity of both acidophilic tannase and gallic acid could be enhanced to about 15-fold under SmF. The process of simultaneous production of acidophilic tannase as an extracellular enzyme along with gallic acid by a marine fungus and their application were reported for the first time. The unique properties of the enzyme rather related to its structure distinguished from the reported terrestrial analogs (Beena et al., 2011).

Thus, the marine laccases, peroxidases, and tannases could be of great interest in both biotechnology and ecology in the cases where a high concentration of salts are required, particularly in the extremely cold environments (Kamei et al., 2008; Raghukumar, 2008; Pang et al., 2011; Bonugli-Santos et al., 2010; Menezes et al., 2010; Feng et al., 2013; Sette and Santos, 2013). Different fungi producing lignolytic enzymes were isolated from polluted marine environments and screened for their possible use in bioremediation (Xue et al., 2015; Deshmukh et al., 2016; Bovio et al., 2017; Barnes et al., 2018). It has been suggested that the marine Ascomycota are major candidate for the decomposition of polyphenol-containing material in seawater and salt marshes, whereas this role in terrestrial environment predominantly belongs to Basidiomycota (Lyons et al., 2003; Panno et al., 2013; Gnavi et al., 2017). One third of the ascomycetes from seawater and sediment sampled in a Mediterranean site continuously contaminated with oil spills was able to grow in the presence of crude oil as the sole carbon source (Bovio et al., 2017). *Aspergillus terreus* MUT 271, *T. harzianum* MUT 290 and *Penicillium citreonigrum* MUT 267 showed a high decolorization percentage (DP ≥ 68%) of 2,6-dichlorophenol indophenol (DCPIP) with the highest decrease of hydrocarbon compounds (up to 40%) for *A. terreus* MUT 271 (Bovio et al., 2017). The redox indicator DCPIP (redox potential +0.217 V) is used for the rapid and simple colorimetric determination of the different types of oil biodegradation profiles for the hydrocarbon-degrading microorganisms based on the decoloration of reduced molecules of the substrate (Bidoia et al., 2010). The oxidation tests with the use of DCPIP allowed selecting the fungi degrading three main fractions of oil in the Reconcavo and Campos Basins: saturated hydrocarbons, aromatic, and non-hydrocarbon compounds (Lima et al., 2017). The isolates of *A. niger*, *Penicillium documbens* and *Cochliobolus lunatus* collected from Pensacola beach (Gulf of Mexico) had the ability to degrade crude oil in the presence of redox indicator, decreasing the hydrocarbon weight approximately by up to 10 % during 7 days (Al-Nasrawi, 2012). Mangrove fungus *Penicillium citrinum* #NIOSN-M126 reduced the total crude oil content by 77% and the individual *n*-alkane fraction by more than 95% (Barnes et al., 2018).

Recently, the full-length or partial sequences of the multigene laccases from the marine-derived fungi with bioremediation potential have appeared in GenBank (Supplementary Table 3). Remarkably, the laccases of the marine strains *Nigrospora* sp. CBMAI 1328 and *Arthopyrenia* sp. CBMAI 1330 have structural features that groups them phylogenetically into the proteins from ascomycetes derived from the marine environments (Passarini et al., 2015). Discovery of the genes involved in the delignification pathways in marine fungi can help to understand their mechanisms to exploit their potential as efficient biomarkers for bioremediation.

## Conclusion

The polysaccharide- and polyphenol-degrading enzymes in marine-derived fungi are often more multitudinous and effective than their terrestrial counterparts, indicating the great contribution of marine fungi to the biotransformation processes of algae and plant material in the ocean parts of the Earth. Many discoveries are expected in the coming years from this yet poorly explored group of microorganisms, particularly about their enzymes specific toward the marine substrates. The alteration of CAZymes in marine fungi caused by the adaptation to marine environment allows them to effectively growth on the algal as well as plant polymeric substrates, including industrial wastes, to produce the mycelium biomass enriched in the proteins and enzymes. Therefore, the gene sequences encoding CAZymes of marine fungi should be explored on their functionality to use in the genetic modification and metabolic improvement of the biotechnological strains, particularly for their cultivation at the high salt concentrations or other extreme conditions in industry or bioremediation of soils and water.

## Author Contributions

LB, OS, and LT reviewed the contents critically. LS drew **Figure 1** and assisted in the preparation of Supplementary Table 1. LB and LS wrote the review.

## Conflict of Interest Statement

The authors declare that the research was conducted in the absence of any commercial or financial relationships that could be construed as a potential conflict of interest.
