# Supplementary material for: Biotechnology Potential of Marine Fungi Degrading Plant and Algae Polymeric Substrates
Source: Front Microbiol. 2018 Jul 10;9:1527. doi: 10.3389/fmicb.2018.01527 (PMC6052901; doi:10.3389/fmicb.2018.01527)
Supplement: Supplementary file 1 [file Table_1.DOCX]

**Table 1. Biomass and carbohydrate-active enzymes production by marine fungi**

| Strain | Sample | Cultivation conditions | Carbon source | Biomass production  (NR- Not reported) | CAZymes production and  specificity  (NR- Not reported) |
| --- | --- | --- | --- | --- | --- |
| Sordariomycetes  *Calcarisporium*sp. KF525  (ITS region ID: KC800713)  (Wang et al., 2016) | German Wadden Sea | Growth medium: 0.5 g L^-1^ KH_2_PO_4_, 0.1 g L^-1^ MgSO_4_ · 7H_2_O, 0.05 g L^-1^ CaCl_2_ ·2H_2_O, 5 mg L^-1^ Citric acid · H_2_O, 5 mg L^-1^ ZnSO_4_ ·7H_2_O, 1 mg L^-1^ Fe(NH_4_)_2_(SO_4_)_2_ · 6H_2_O, 50 μg L^-1^ MnSO_4_ · 4H_2_O, 260 μg L^-1^ CuSO_4_ · 5H_2_O, 50 μg L^-1^ H_3_BO_3_, 50 μg L^-1^ Na_2_MoO_4_ · 2H_2_O, and 50 μg L^-1^ biotin (pH 5.5), 2g L**^-^**^1^ carbohydrate; 7 days, 10 mL culture, 20 °C, 180 rpm | glucose  arabinogalactan  agar  alginate  carragenan  laminarin  ulvan  fucoidan | 0.47 ± 0.02 g L^-1^  0.26 ±0.05 g L^-1^  0.12 ± 0.02 g L^-1^  0.12 ±0.01 g L^-1^  0.12 ±0.01 g L^-1^  0.11 ± 0.01 g L^-1^  0.08 ± 0.01 g L^-1^  ≤0.08 ± 0.01 g L^-1^ | Substrate specificity: laminarin ≤ glucose ≤ galactose ≤ fucose ≤ fucoidan ≤ cellulose ≤ pectin ≤ starch ≤ xylan birch ≤ arabinose ≤ alginate ≤ agar ≤ carrageenan ≤ CMC ≤ xylan *Undaria* ≤ ara-galactan ≤ ulvan E ≤ ulvan U (the colony radial growth rate from 13 to 35 µm h^-1^) |
| Sordariomycetes  *Tritirachium*sp. LF562  (ITS region ID: FR822828)  (Wang et al., 2016) | Sponge, the Adriatic Sea | Growth medium: 0.5 g L^-1^ KH_2_PO_4_, 0.1 g L^-1^ MgSO_4_ · 7H_2_O, 0.05 g L^-1^ CaCl_2_ ·2H_2_O, 5 mg L^-1^ Citric acid · H_2_O, 5 mg L^-1^ ZnSO_4_ ·7H_2_O, 1 mg L^-1^ Fe(NH_4_)_2_(SO_4_)_2_ · 6H_2_O, 50 μg L^-1^ MnSO_4_ · 4H_2_O, 260 μg L^-1^ CuSO_4_ · 5H_2_O, 50 μg L^-1^ H_3_BO_3_, 50 μg L^-1^ Na_2_MoO_4_ · 2H_2_O, and 50 μg L^-1^ biotin (pH 5.5); 2g L**^-^**^1^ carbohydrate; 7 days, 10 mL culture, 30 °C, 180 rpm | glucose  laminarin  arabinogalactan  alginate  ulvans  fucoidan  agar  carragenan | 0.57 g/1g substrate (S)  0.57 g/1g S  0.35 ± 0.25 g L^-1^  1.04 ± 0.19 g L^-1^  0.12 ± 0.05 g L^-1^  ≤ 0.01 ± 0.01 g L^-1^  ≤ 0.01 ± 0.01 g L^-1^  ≤0.01 ± 0.01 g L^-1^ | Substrate specificity: fucoidan ≤ ulvan E ≤fucose ≤ xylan birch ≤ laminarin ≤ pectin ≤ starch ≤ arabinose ≤ ara-galactan ≤ glucose ≤ galactose ≤ agar ≤ CMC ≤ carrageenan ≤ ulvan U ≤ cellulose ≤ xylan *Undaria* ≤ alginate (the colony radial growth rate from 20 to 55 µm h^-1^) |
| Sordariomycetes  *Bartalinia robillardoides*LF550  (ITS region ID: FR822822)  (Wang et al., 2016) | Sponge *Tethya aurantium*, the Adriatic Sea | Growth medium: 0.5 g L^-1^ KH_2_PO_4_, 0.1 g L^-1^ MgSO_4_ · 7H_2_O, 0.05 g L^-1^ CaCl_2_ ·2H_2_O, 5 mg L^-1^ Citric acid · H_2_O, 5 mg L^-1^ ZnSO_4_ ·7H_2_O, 1 mg L^-1^ Fe(NH_4_)_2_(SO_4_)_2_ · 6H_2_O, 50 μg L^-1^ MnSO_4_ · 4H_2_O, 260 μg L^-1^ CuSO_4_ · 5H_2_O, 50 μg L^-1^ H_3_BO_3_, 50 μg L^-1^ Na_2_MoO_4_ · 2H_2_O, and 50 μg L^-1^ biotin (pH 5.5); 2g L**^-^**^1^ carbohydrate; 7 days, 10 mL culture, 30 °C, 180 rpm | glucose  laminarin  arabinogalactan  ulvans  fucoidan  agar  carrageenan  alginate | ≤ 1.6± 0.05 g L^-1^  ≥1.4± 0.05 g L^-1^  0.11 ± 0.04 g L^-1^  0.07/0.08 ± 0.04 g L^-1^  ≤ 0.01 ± 0.01 g L^-1^  ≤ 0.01 ± 0.01 g L^-1^  ≤ 0.01 ± 0.01 g L^-1^  - | Substrate specificity: fucoidan ≤ fucose ≤ galactose ≤ xylan birch ≤ starch ≤ cellulose ≤ arabinose ≤ glucose ≤ carrageenan ≤ alginate ≤ pectin ≤ CMC ≤ agar ≤ ara-galactan ≤ xylan Undaria ≤ ulvan U ≤ ulvan E ≤ laminarin (the colony radial growth rate from 42 to 140 µm h^-1^) |
| Eurotiomycetes  *Penicillium pinophilum*LF458  (Wang et al., 2016) | Marine environment | Growth medium: 0.5 g L^-1^ KH_2_PO_4_, 0.1 g L^-1^ MgSO_4_ · 7H_2_O, 0.05 g L^-1^ CaCl_2_ ·2H_2_O, 5 mg L^-1^ Citric acid · H_2_O, 5 mg L^-1^ ZnSO_4_ ·7H_2_O, 1 mg L^-1^ Fe(NH_4_)_2_(SO_4_)_2_ · 6H_2_O, 50 μg L^-1^ MnSO_4_ · 4H_2_O, 260 μg L^-1^ CuSO_4_ · 5H_2_O, 50 μg L^-1^ H_3_BO_3_, 50 μg L^-1^ Na_2_MoO_4_ · 2H_2_O, and 50 μg L^-1^ biotin (pH 5.5); 2g L**^-^**^1^ carbohydrate; 7 days, 10 mL culture, 30 °C, 180 rpm | laminarin  glucose  alginate  agar  fucoidan  arabinogalactan  ulvans  carragenan | 0.36 ± 0.05 g L^-1^  0.29 ± 0.06 g L^-1^  ≤ 0.35± 0.25 g L^-1^  ≤ 0.25± 0.25 g L^-1^  ≤ 0.01± 0.01 g L^-1^  ≤0.01 ± 0.01 g L^-1^  ≤0.01 ± 0.01 g L^-1^  - | Substrate specificity: arabinose ≤ fucoidan ≤ alginate ≤ carrageenan ≤ cellulose ≤ agar ≤ ara-galactan ≤ fucose ≤ galactose ≤ ulvan U ≤ xylan birch ≤ CMC ≤ pectin ≤ starch glucose ≤ xylan Undaria ≤ ulvan E ≤ laminarin (the colony radial growth rate from 100 to 180 µm h^-1^) |
| Sordariomycetes  *Pestalotiopsis* sp. KF079  (Taxonomy ID*:* 1938903)  (Wang et al., 2016) | Marine environment, mangrove endophyte or saprophyte | Growth medium: 0.5 g L^-1^ KH_2_PO_4_, 0.1 g L^-1^ MgSO_4_ · 7H_2_O, 0.05 g L^-1^ CaCl_2_ ·2H_2_O, 5 mg L^-1^ Citric acid · H_2_O, 5 mg L^-1^ ZnSO_4_ ·7H_2_O, 1 mg L^-1^ Fe(NH_4_)_2_(SO_4_)_2_ · 6H_2_O, 50 μg L^-1^ MnSO_4_ · 4H_2_O, 260 μg L^-1^ CuSO_4_ · 5H_2_O, 50 μg L^-1^ H_3_BO_3_, 50 μg L^-1^ Na_2_MoO_4_ · 2H_2_O, and 50 μg L^-1^ biotin (pH 5.5); 2g L**^-^**^1^ carbohydrate; 7 days, 10 mL culture, 30 °C, 180 rpm | laminarin  arabinogalactan  fucoidan  ulvans  agar  carragenan  alginate  glucose | 0.48±0.06 g L^-1^  0.18±0.07 g L^-1^  0.08 g L^-1^  0.05±0.00 g L^-1^  0.03± 0.01 g L^-1^  ≤ 0.01 ± 0.01 g L^-1^  -  - | Substrate specificity: galactose ≤ arabinose ≤ agar ≤ cellulose ≤ fucose ≤ CMC ≤ carrageenan ≤ alginate ≤ fucoidan ≤ xylan birch ≤ ulvan U ≤ xylan Undaria ≤ galactan ≤ ulvan E ≤ pectin ≤ laminarin ≤ starch ≤ glucose (the colony radial growth rate from 150 to 380 µm h^-1^) |
| Sordariomycetes  *Scopulariopsis brevicaulis*LF580  (18S rRNA ID: KM096194)  (Wang et al., 2016)  *Scopulariopsis brevicaulis*LF580  (BioSample accession ID: SAMN03764504) (Kumar et al., 2015) | Sponge *Tethya aurantium*, the Adriatic Sea | Growth medium: 0.5 g L^-1^ KH_2_PO_4_, 0.1 g L^-1^ MgSO_4_ · 7H_2_O, 0.05 g L^-1^ CaCl_2_ ·2H_2_O, 5 mg L^-1^ Citric acid · H_2_O, 5 mg L^-1^ ZnSO_4_ ·7H_2_O, 1 mg L^-1^ Fe(NH_4_)_2_(SO_4_)_2_ · 6H_2_O, 50 μg L^-1^ MnSO_4_ · 4H_2_O, 260 μg L^-1^ CuSO_4_ · 5H_2_O, 50 μg L^-1^ H_3_BO_3_, 50 μg L^-1^ Na_2_MoO_4_ · 2H_2_O, and 50 μg L^-1^ biotin (pH 5.5); 2g L**^-^**^1^ carbohydrate; 7 days, 10 mL culture, 30 °C, 180 rpm  Growth medium:1% glucose×H_2_O, 0.5% soy peptone, 0.3% malt extract, 0.3% yeast extract, 3% NaCl, 2% agar;  4 days, 100 mL culture, 200 rpm in the dark, 28 C | glucose  laminarin  arabinogalactan  alginate  ulvans  fucoidan  agar  carragenan  1% glucose, 2% agar, 0.3% malt extract | 1.00±0.01 g L^-1^ (0.49 g/1g)  0.97±0.03 g L^-1^ (0.49 g/1g)  0.3±0.01 g L^-1^  0.25±0.05 g L^-1^  0.15/0.23±0.04 g L^-1^  ≤0.15±0.04 g L^-1^  ≤0.15±0.04 g L^-1^  ≤0.15±0.04 g L^-1^  5.7 ± 0.9 mg(dry biomass)mL^-1^ | Substrate specificity: glucose ≤ fucose ≤ galactose ≤ pectin ≤ laminarin ≤ starch ≤ agar ≤ arabinose ≤ fucoidan ≤ ulvan E ≤carrageenan ≤ CMC ≤ alginate cellulose ≤ xylan birch ≤ ara-galactan ≤ xylan *Undaria* ≤ ulvan U (the colony radial growth rate from 30 to 270 µm h^-1^)  NR |
| Eurotiomycetes  *Aspergillus oryzae* RP1  (ITS region ID: GU385811)  (Singh et al., 2011) | Brown alga seaweed *Dictyota dichotoma,* Gulf of Kutch (India) | Basal salt medium: 3% NaCl, 0.2% KCl, 0.01% MgSO4, 0.01% yeast extract, and 0.05% peptone (w/v); 4 days, 100 mL, 35±1°C, pH 6.5, 150rpm | 2% sodium alginate | 18 mg (total potein) mL^-1^  (completely lost activity at 50°C) | alginate lyase (21.11% yield after purification);  67.24 U/mg protein (polyM, polyG blocks of alginate) |
| Sordariomycetes  *Arthrinium saccharicola*KUC21221 (beta-tubulin ID: AKT73471)  (Hong et al., 2015) | Marine brown algae *Sargassum* sp., Hyeopjae beach, Korea | Mandels’ medium, per liter: 0.3 g urea, 1.4 g KH_2_PO_4_, 2.0 g (NH_4_)_2_SO_4_, 0.3 g CaCl_2_, 0.3 g MgSO_4_, 0.25 g yeast extract, 0.75 g peptone, 5 mg FeSO_4_·7H_2_O, 36 mg OCl_2_·6H_2_O, 1.8 mg MnSO_4_·H_2_O, 2.5 mg ZnSO_4_·7H_2_O; 7 days, 10 mL, 25 °C, 150 rpm | 1% cellulose | NR | endoglucanase (EG): 0.39± 0.13 U/mL (FPU);  0.38± 0 U/mL (CMC)  β-glucosidase (BGL): 1.04±0.03 U/mL (pNPG)  β-xylosidase (BXL): 0.02 ± 0 U/mL (pNPX) |
| Sordariomycetes  *Diaporthe arecae* KUC21217  (beta-tubulin ID: AKT73467)  (Hong et al., 2015) | Marine brown algae *Sargassum* sp., Hyeopjae beach, Korea | Mandels’ medium, per liter: 0.3 g urea, 1.4 g KH_2_PO_4_, 2.0 g (NH_4_)_2_SO_4_, 0.3 g CaCl_2_, 0.3 g MgSO_4_, 0.25 g yeast extract, 0.75 g peptone, 5 mg FeSO_4_·7H_2_O, 36 mg OCl_2_·6H_2_O, 1.8 mg MnSO_4_·H_2_O, 2.5 mg ZnSO_4_·7H_2_O; 7 days, 10 mL culture, 25 °C, 150 rpm | 1% cellulose | NR | endoglucanase (EG): 0.16 ± 0.01 U/mL (FPU);  0.14 ± 0.03 U/mL (CMC)  β-glucosidase (BGL): 0.29 ± 0.08 U/mL (pNPG)  β-xylosidase (BXL): 0.01 ± 0.01 U/mL (pNPX) |
| Eurotiomycetes  *Penicillium* sp. PDA2  (18S rRNA ID: FJ538201)  (Baker et al., 2010)  *Hypocrea jecorina* QM9414 *(T. resee)* (Baker et al., 2010) | Sponge *Haliclona simulans*, Gurraig Sound Kilkieran Bay, Galway, the west coast of Ireland  cellulase overproducing mutant | Growth medium: 5 g L^-1^ CMC, 1 g L^-1^ peptone, 1.2 g L^-1^ KH_2_PO_4_, 0.5 g L^-1^ Mg_2_SO_4_, 0.05 g L^-1^ FeCl_3_ (5% (w⁄ v) NaCl); 14 days, 100 ml culture, 28 °C, 150 rpm  - | 5 g L^-1^ CMC  - | NR  NR | endoglucanase (EG): 15 U/mg protein(CMC, 50°C);  0.34 ± 0.003 U/mg protein (FPU, 50°C)  endoglucanase (EG): ≤10 U/mg protein (CMC, 50°C);  0.31 ± 0.14 U/mg protein (FPU, 50°C) |
| Sordariomycetes  *Engyodontium album* TISTR 3645  (Baker et al., 2010) | Solar saltern, Thailand | Growth medium: 8.0 g L^-1^ CaCO_3_, 0.15 g L^-1^ FeSO_4_·7H_2_O, 3.5 g L^-1^ KH_2_PO_4_, 0.10 g L^-1^ MgSO_4_·7H_2_O, 3.0 g L^-1^ mycological peptone, 6.6 g L^-1^(NH_4_)2SO_4_ | 10 g L^-1^ starch | NR | α-amylase, 50 kDa: 132.17 U/mg protein (pH 9.0, 60ºC, 30% NaCl) |
| Eurotiomycetes  *Aspergillus oryzae* (Sathya et al., 2013) | Marine sediments, the east coast of India | Growth medium: potato dextrose broth, 5 g of spoiled banana fruit powdered with the mineral salt medium: 2.2% Na_2_HPO_4_·2H_2_O, 1.22% Na_2_HPO_4_x2H_2_O, 0.6% KCl, 0.02% MgSO_4_x7H_2_O;  50 mL, 35-40 °C, pH 6.5, 96 h | 100 g L^-1^ spoiled banana fruit powder with supplementation of starch | NR | α-amylase: 250 U/mg protein (DNSA method) |
| Eurotiomycetes  *Penicillium* sp. NIOM-02 (Dhale et al., 2009) | Marine sediments, Miramar, India. | Growth medium: peptone 10 g L^-1^; yeast extract 5 g L^-1^, corn flour 20 g L^-1^;  50 mL, 10 days, pH 5.5, 118 rpm | corn flour 20 g L^-1^ | NR | α-amylase, 53-kDa: 246.5 U/mg protein (37 ºC) |
| Dothideomycetes  *Cladosporium sphaerospermum* (Trivedi et al., 2015) | Marine environment, India | *U. fasciata* seaweed biomass;  SSF, 60% moisture, 4 days, 25°C, pH 4 | *Ulva* seaweed polysaccharides | NR | endoglucanase (EG): 10.20 ± 0.40 U/g dry weight (CMC);  9.60 ± 0.64 U/g dry weight (FPU);  Yield of sugar: 112 ± 10 mg/g dry weight, yield of bioethanol: 0.47 g/g dry weight (10 U/g enzymes, 24 h, 40 ºC, pH 4) |
| Sordariomycetes  *Trichoderma hamatum* FU21 (ITS region ID: KP836330)  (Lee et al., 2015) | Algae *Agarum cribrosum*, the eastern coast of Korea | Mandels’ medium, per liter: 0.3 g urea, 1.4 g KH_2_PO_4_, 2.0 g (NH_4_)_2_SO_4_, 0.3 g CaCl_2_, 0.3 g MgSO_4_, 0.25 g yeast extract, 0.75 g peptone, 5 mg FeSO_4_·7H_2_O, 36 mg OCl_2_·6H_2_O, 1.8 mg MnSO_4_·H_2_O, 2.5 mg ZnSO_4_·7H_2_O;  7 days, 25°C, 150 rpm | 1% cellulose | 0.163 ± 0.019 mg/mL (total protein) | endoglucanase (EG): 0.568 ± 0.073; 0.209 ± 0.03 U/mL (FPU)  β-glucosidase (BGL): 0.204 ± 0.025 U/mL |
| Eurotiomycetes  *Aspergillus* cf. *tubingensis* LAMAI 31 (beta-tubulin ID: KT935448)  (Dos Santos et al., 2016) | Sponge *Dragmacidon reticulatum*, the coastline of Brazil | Mandels and Sternbergs medium (MS), containing in g^.^L^-1^: 1.0 peptone, 1.4 (NH_4_)_2_SO_4_, 2.0 KH_2_PO_4_, 0.3 urea, 0.3 CaCl_2_, 0.3 MgSO_4_^.^7H_2_O, 0.005 FeSO_4_^.^7H_2_O, 0.001 MnSO_4_^.^H_2_O, 0.001 ZnSO_4_^.^7H_2_O, 0.002 CoCl_2_, pH 6.0;  50 mL, 7 days, 140 rpm and 28 °C | 90 g L^−1^ rice straw,  60 g L^−1^ sugarcane bagasse | NR | Xylanase: 561.59 U/mL (55 ºC, pH 5) |
| Dothideomycetes  *Cladosporium*sp  (Del-Cid et al., 2014) | Antarctic marine sponges | Czapek minimal medium;  7 days, 15-23 ºC | 1% beechwood or birchwood xylan, or wheat bran | NR | Xylanase activity (50 ºC, pH 6) determination by staining the plates |
| Sordariomycetes  *Trichoderma harzianum* CKP01; (ITS region ID: KC330218); (Thirunavukkarasu et al., 2015) | Algae *Sargassum wightii* | BX medium per liter: 5g yeast extract, 1g NaNO_3_, 1g KH_2_PO_4_, 1g peptone, 0.3g MgSO_4_⋅7H_2_O;  5 days, pH 5.5, 120 rpm, 31 °C | 9 g L^−1^ oat spelt xylan (Sigma) | NR | Xylanase: 2.2 μmoL/min/mg protein  Xylosidase: 0.0024 μmoL/min/mg protein;  1.5-2% NaCl increased activities by twofold) |
| Eurotiomycetes  *Penicillium* sp. FS010  (*Penicillium chrysogenum*A3969.2 18S rRNA ID: AY593254)  (Hou et al., 2006) | Yellow Sea sediments | Recombinant *E.coli* BL21 medium | - | - | Xylanase GH10: 1868 U/mg (25 °C, pH 5.5) - endo-1,4-beta-D-xylan xylanohydrolase: GenBank ID: DQ304546; Xylosidase: <10^−2^ U/mg; Exo-cellobiohydrolase (CBHI) GH7: GenBank ID: AY973993 |
| Eurotiomycetes  *Aspergillus niger* NIOCC  (Raghukumar et al., 2004) | Mangrove detritus | Basal medium on sea water per liter: 2 g KH_2_PO_4_, 1.45 g MgSO_4_·7H_2_O, 0.132 g CaCl_2_·2H_2_O, 1 mg thiamine-HCl, 28 ml 1.2 mM ammonium tartrate;  20 mL, 7 days, 30°C, pH8.5 | 1% oat spelt xylan | 157 mg (dry weight) | Xylanase: 48.8 U/L= 3.8 U/mg (2457 U/mg after purification) (pH 3.5, 50°C; pH 8.5, 80°C)  β-d-Xylosidase: 260 U/L  α-l-arabinofuranosidase: 50 U/L  cellulase: 0 U/L (CMC, FPU) |
| Sordariomycetes  *Fusarium moniliforme*NCIM 1276 (Niturea et al., 2008) | Mangrove detritus, the west coast of India | 37.9 mM (NH_4_)_2_SO_4_, 11.5 mM K_2_HPO_4_, 14.7 mM KH_2_PO_4_ and 6.8 mM CaCl_2_ with 1% sugar;  50 mL, pH5-8, 30 °C, 200 rpm, 96 h | 1% galacturonic acid, polygalacturonic acid, pectin (8% esterified), glucose, starch, xylan, cellulose and wheat bran | 315.5±3.2 mg (maximal biomass production at pH6) | PG: 11.5±0.08 U (pectin,pH5); 2.5±0.13 U (pH8)  PL: 26±0.12 U (pectin,pH5); 180±0.51 U (pH8)  CMCase: 2.5±0.13 U (Wheat bran, pH5); 9±1.1 U (pH8)  Xylanase: 28.7±0.18 (Wheat bran, pH5); 60±3.1 U (pH8)  Amilase: 16±1.5 (Wheat bran, pH5); 18.4±0.1 (pH8) |
| *Pestalotiopsis* sp. J63 (Feng et al., 2013) | Marine sediments,  the East China Sea | 2 g·L^-1^ ammonium tartrate, 5 g·L^-1^ maltose and 5 g·L^-1^ rice straw;  50 mL, 28 °C, 160 rpm, 5 days | 5 g·L^-1^ maltose and  5 g·L^-1^ rice straw | 0.5 mg·mL^-1^ (for 2 days of cultivation) | Laccase: 666.3 ± 27.7 U·L^-1^;  5791.7 U·L^-1^ (0.09 mmol·L^-1^ phenol as inductor at 24 h of cultivation) |
| *Mucor racemosus* CBMAI 847  (Bonugli-Santos et al., 2010)  ITS-rDNA ID: FJ790879 | Northern coast, São Paulo, Brazil | 0.075 g yeast extract; 0.025 g NH4Cl;  6.25 g NaCl;  50 ml, 28 ◦C, 140 rpm, 15 days | 0.5 g glucose,  2.4 mg·mL^-1^wheat bran |  | MnP: 4484.30 IU·L^-1^ (12.5% (w/v) salinity)  Laccase: 898.15 UI·L^-1^ (23% (w/v) salinity) |
| Sordariomycetes  *Alternaria alternate*  (Faten et al., 2013) | Decayed wood of old fishing boats from Ismalia, Egypt | 3g of plant substrate, 15 ml seawater for SSF; or 50 ml of seawater,  150 rpm for SMF  25-28 ºC, 250 ml flask, 21 days | wheat bran |  | CMCase 9.68 U/g  Xylanase 24.84 U/g  Laccase 1721.43 U/g Pectinase 97.08 U/g |
