# Supplementary material for: Biotechnology Potential of Marine Fungi Degrading Plant and Algae Polymeric Substrates
Source: Front Microbiol. 2018 Jul 10;9:1527. doi: 10.3389/fmicb.2018.01527 (PMC6052901; doi:10.3389/fmicb.2018.01527)
Supplement: Supplementary file 2 [file Table_2.DOCX]

**Table 2a. Putative plant-polysaccharide degrading activities in genome-sequenced marine fungi**

| **Substrate** | **Enzyme** | **EC no., Abbreviation** | **CAZyme families*** | ***Scopulariopsis brevicaulis*** LF580 (Kumar et al., 2015) | ***Pestalotiopsis* sp. NCi6**  (Arfi et al., 2013) |
| --- | --- | --- | --- | --- | --- |
| Cellulose | β-1,4-Endoglucanase  exo β-glucanase (cellobiohydrolase, reducing end)  exo β-glucanase Cellobiohydrolase (nonreducing end)  β-1,4-Glucosidase  Cellobiose dehydrogenase  Lytic polysaccharide monooxygenase | EG, 3.2.1.4  CBHI, 3.2.1.176  CBHII, 3.2.1.91  BGL, 3.2.1.21  CDH, 1.1.99.18  LPMO | GH3, -5, -6, -7, -9, -12, -45  GH7  GH6  GH1, -3  AA3_1, AA7, AA8  AA9 (formerly GH61) | GH3, -5, -6, -7, -12, -45  GH7  GH6  GH1, -3  AA3_1; AA7; AA8  AA9 | GH3, -5, -6, -7  GH7  GH6  GH3  AA3_1; AA8 (only in saline medium)  - |
| Xylan  Galactomannan  Xyloglucan  Arabinoxylan | β-1,4-Endoxylanase.  β-1,4-Xylosidase  β-1,4-Endomannanase  β-1,4-Mannosidase  β-1,4-Galactosidase  α-1,4-Galactosidase  α-Arabinofuranosidase  Xyloglucan β-1,4-endoglucanase  α-Xylosidase  α-Fucosidase  α-1,4-Galactosidase  β-1,4-Galactosidase  Arabinoxylan arabinofuranohydrolase  /arabinofuranosidase  α-Glucuronidase  α-1,4-Galactosidase  β-1,4-Galactosidase  Acetyl xylan esterase  Feruloyl esterase | XLN, 3.2.1.8  BXL, 3.2.1.37  MAN, 3.2.1.78  MND, 3.2.1.25  LAC, 3.2.1.23  AGL, 3.2.1.22  ABF, 3.2.1.55  XEG, 3.2.1.151  AXL, 3.2.1.177  AFC, 3.2.1.51  AGL, 3.2.1.22  LAC, 3.2.1.23  AXH, 3.2.1.55  AGU, 3.2.1.139  AGL, 3.2.1.22  LAC, 3.2.1.23  AXE, 3.1.1.72  FAE, 3.1.1.73 | GH10, -11  GH3, -43  GH5, -26  GH2  GH2, -35  GH27, -36  GH51, -54  GH12, -74  GH31  GH29, -95  GH27, -36  GH2, -35  GH62  GH67, -115  GH27, -36  GH2, -35  CE1, -5  CE1 | GH10, -11  GH3, -43  GH5, -26  GH2  GH2, -35  GH27, -36  GH51, -54  GH12, -74  GH31  GH95  GH27, -36  GH2, -35  GH62  GH67, -115  GH27, -36  GH2, -35  CE1, -5  CE1 | GH10, -11  GH3, -43  GH5  GH2  GH2, -35  GH27, -36  GH51  GH74  GH31  GH29  GH27, -36  GH2, -35  GH62  GH115 (only in saline medium)  GH27, -36  GH2, -35  CE1  CE1 |
| Pectin  Starch | Endopolygalacturonases  Exopolygalacturonases  Endorhamnogalacturonase  Exorhamnogalacturonase  Rhamnogalacturonan rhamnohydrolase  α-Rhamnosidase  α-Arabinofuranosidase  Endoarabinanase  Exoarabinanase  β-1,4-Endogalactanase  Unsaturated glucuronyl hydrolase  Unsaturated rhamnogalacturonan hydrolase  β-1,4-Xylosidase  β-1,4-Galactosidase  Pectin lyase  Pectate lyase  Rhamnogalacturonan lyase  Pectin methyl esterase  Rhamnogalacturonan acetyl esterase  Feruloyl esterase  α-Amylase  Glucoamylase  α-1,4-Glucosidase | PGA, 3.2.1.15  PGX, 3.2.1.67  RHG, 3.2.1.171  RHX, 3.2.1.-  RGXB, 3.2.1.174  RHA, 3.2.1.40  ABF, 3.2.1.55  ABN, 3.2.1.99  ABX, 3.2.1.–  GAL, 3.2.1.89  UGH, 3.2.1.–  URH, 3.2.1.172  BXL, 3.2.1.37  LAC, 3.2.1.23  PEL, 4.2.2.10  PLY, 4.2.2.2  RGL, 4.2.2.23  PME, 3.1.1.11  RGAE, 3.1.1.–  FAE, 3.1.1.73  AMY, 3.2.1.1  GLA, 3.2.1.3  AGD, 3.2.1.20 | GH28  GH28  GH28  GH28  GH28  GH78  GH51, -54, -62  GH43  GH93  GH53  GH88  GH105  GH3, -43  GH2, -35  PL1  PL1, -3, -9  PL4, -11  CE8  CE12  CE1  GH13  GH15  GH31 | GH28  GH28  GH28  GH28  GH28  GH78  GH51, -54, -62  GH43  GH93  GH53  GH88  GH105  GH3, -43  GH2, -35  PL1  PL1, -3, -9  PL4, -11  CE8  CE12  CE1  GH13  GH15-CMB20  GH31 | GH28(only in saline medium)  GH28  GH28  GH28  GH28  GH78  GH51  GH43  GH93  GH53  -  GH105  GH3, -43  GH2, -35  PL1  PL1, -3  PL4  CE8  CE12  CE1  GH13  GH15  GH31 |

* CAZy families and relative activities were taken from van den Brink et al., 2011; Rytioja et al., 2014; Zhao et al., 2014; Berlemont etal., 2017
