# Supplementary material for: Biotechnology Potential of Marine Fungi Degrading Plant and Algae Polymeric Substrates
Source: Front Microbiol. 2018 Jul 10;9:1527. doi: 10.3389/fmicb.2018.01527 (PMC6052901; doi:10.3389/fmicb.2018.01527)
Supplement: Supplementary file 3 [file Table_3.DOCX]

**Table 2b. Putative algae-polysaccharide degrading activities in genome-sequenced marine fungi**

| **Substrate** | **Enzyme** | **EC no.** | **CAZyme families** | ***Scopulariopsis brevicaulis* LF580** | ***Pestalotiopsis* sp. NCi6** |
| --- | --- | --- | --- | --- | --- |
| Agarose | β-Agarase  α-Agarase | 3.2.1.81  3.2.1.- | GH16, -50, -86, -118  GH117 | GH16  - | GH16  - |
| Alginate  Oligo alginate | Alginate lyase  Oligo alginate lyase | 4.2.2.3  4.2.2.- | PL7, -17  PL15 | -  - | -  - |
| Carrageenan | κ- Carrageenase  ι-Carrageenase  λ-Carrageenase | [3.2.1.83](http://www.enzyme-database.org/query.php?ec=3.2.1.83) | GH16  GH82  PL11 | GH16  -  PL11 | GH16  -  - |
| Laminarin, lichenin, (1,3)- or (1–4)-β-D-glucosidic linkages  Laminarin, (1–3)-α-D-glucosidic linkages  Laminarin, (1–6)-α-D-glucosidic linkages  Laminarin, (1–6)- α-D-glucosidic linkages  Laminarin, lichenan, pustulan and cellulosic derivatives, (1-3)- and (1-6) or (1-4)-β-D-glucosidic linkages | Endo-beta-1,3-1,4 glucanase (Licheninase)  Beta-1,3-glucanase  Beta-1,6-glucanase  Pullulanase  Beta-glucanase  (exo-β-1,3/1,6- and endo-β-1,4-glucanase) | [3.2.1.6](http://www.enzyme-database.org/query.php?ec=3.2.1.6)  [3.2.1.73](http://www.enzyme-database.org/query.php?ec=3.2.1.73)  [3.2.1.39](http://www.enzyme-database.org/query.php?ec=3.2.1.39)  3.2.1.75  3.2.1.41  - | GH3,16,26  GH16, -17, -55, -64, -81  GH5, -13, -30  GH13  GH131 | GH3,16,26  GH16, -17, -55, -81  GH5, -13, -30  GH13  GH131 | GH3,16  GH16, -17, -55, -64, -81  GH5, -13, -30  GH13  - |
| Fucan, (1-3), or (1-4)-α-L-fucan;  (1-3), or (1-4)-α-L-fucan  (1-2)-α-L-fucan | Fucoidanase  α-L-Fucosidase; α-1,3/1,4-L-Fucosidase  α-1,2-L-Fucosidase | 3.2.1.44  [3.2.1.51](http://www.enzyme-database.org/query.php?ec=3.2.1.51)  [3.2.1.111](http://www.enzyme-database.org/query.php?ec=3.2.1.111)  [3.2.1.63](http://www.enzyme-database.org/query.php?ec=3.2.1.63) | GH107  GH29  GH95 | -  -  GH95 | -  -  - |
| Ulvan, (1-4)-β-glucuronic acids | Ulvan lyase | 4.2.2.- | PL24, -25 | - | - |
|  | Unsaturated β-glucuronyl hydrolase | 3.2.1.172 | GH105 | GH105 | GH105 |

*CAZy families and relative activities were taken from Sova et al., 2013; Pluvinage et al., 2013; Collén et al., 2014; Kusaykin et al., 2016; Zhu et al., 2016; Mai et al., 2016; Gao et al., 2017; Ulaganathan et al., 2017
