# Supplementary material for: Biotechnology Potential of Marine Fungi Degrading Plant and Algae Polymeric Substrates
Source: Front Microbiol. 2018 Jul 10;9:1527. doi: 10.3389/fmicb.2018.01527 (PMC6052901; doi:10.3389/fmicb.2018.01527)
Supplement: Supplementary file 4 [file Table_4.DOCX]

**Table 3. Multigene laccase sequences of marine fungi**

| Source | Partial length, amino acid residues (aa) | Gene Bank ID | Reference |
| --- | --- | --- | --- |
| marine ascomycete SAP142 | 308 | AAN17298  Submitted:  24-JUL-2016 | Lyons, et al., 2003 |
| marine ascomycete SAP162 | 303 | AAN17287 | Lyons, et al., 2003 |
| *Lachnum spartinae*  Ascomycota, Leotiomycetes | 259 | AAN17302 | Lyons, et al., 2003 |
| *Panorbis viscosus*  Ascomycota, Sordariomycetes | 302 | AAN17301 | Lyons, et al., 2003 |
| *Phaeosphaeria halima*  Ascomycota, Dothideomycetes | 311  309  295  294  302  300 | AAN17300  AAN17299  AAN17291  AAN17292  AAN17286  AAN17285 | Lyons, et al., 2003 |
| *Pleospora spartinae* | 308 | AAN17297 | Lyons, et al., 2003 |
| *Buergenerula spartinae*  Ascomycota, Sordariomycetes | 310 | AAN17296 | Lyons, et al., 2003 |
| *Mycosphaerella* sp. 2 SAP154  *Mycosphaerella* sp. 2 SAP136  *Mycosphaerella* sp. 2 SAP133  Ascomycota, Dothideomycetes | 300  301  300 | AAN17295  AAN17294  AAN17293 | Lyons, et al., 2003 |
| *Phaeosphaeria spartinicola*  Ascomycota, Dothideomycetes | 260  300  302  302 | AAN17290  AAN17284  AAN17283  AAN17282 | Lyons, et al., 2003 |
| *Stagonospora* sp. SAP143  Ascomycota, Dothideomycetes | 306  287 | AAN17289  AAN17288 | Lyons, et al., 2003 |
| *Tinctoporellus* sp. CBMAI 1061  Basidiomycota, Agaricomycetes | 51  51  51  53 | ADD54658  ADD54657  ADD54656  ADD54655  Submitted:  25-JUL-2016 | Bonugli-Santos, et al., unpublished |
| *Peniophora* sp. CBMAI 1063  Basidiomycota, Agaricomycetes | 51  50 | ADD54654  ADD54653 | Bonugli-Santos, et al., unpublished |
| *Marasmiellus* sp. CBMAI 1062  Basidiomycota, Agaricomycetes | 51  50  51  61 | ADD54651  ADD54650  ADD54649  ADD54652 | Bonugli-Santos, et al., unpublished |
| *Arthopyrenia* sp. CBMAI 1330  Ascomycota, Dothideomycetes | 129 | AJZ68867  Submitted:  29-MAR-2015 | Passarini, et al., unpublished |
| *Peniophora* sp. CBMAI 1063  Basidiomycota,  Agaricomycetes | 552  588  522  482  514  528  531  548  517  517 | AUK50753  AUK50752  AUK50751  AUK50750  AUK50749  AUK50748  AUK50747  AUK50746  AUK50745  AUK50744  Submitted:  09-JAN-2018 | Otero, et al., 2017 |
| *Cerrena unicolor*  Basidiomycota, Agaricomycetes | 47 | P85430  Submitted:  25-OCT-2017 | D'Souza-Ticlo, et al., 2009 |
